# Virus-Induced Risk of Hepatocellular Carcinoma: Recent Progress and Future Challenges

**DOI:** 10.3390/jcm11010208

**Published:** 2021-12-31

**Authors:** Joachim Lupberger, Thomas F. Baumert

**Affiliations:** 1 Institut National de la Santé et de la Recherche Médicale, U1110, 67000 Strasbourg, France; 2 Institut de Recherche sur les Maladies Virales et Hépatiques, Université de Strasbourg, 67000 Strasbourg, France; 3 Institut Hospitalo-Universitaire, Pôle Hépato-Digestif, Hôpitaux Universitaires de Strasbourg, 67091 Strasbourg, France; 4 Institut Universitaire de France, 75231 Paris, France

Chronic viral hepatitis is a key risk factor for liver fibrosis and hepatocellular carcinoma (HCC) [1]. Although the clinical course of virus-induced liver disease is similar in chronic hepatitis B and C, the mechanisms and challenges of disease management are distinct. While an efficient preventive hepatitis B virus (HBV) vaccine is available, chronic HBV infection can only be controlled by the current standard of care and is rarely cured. The contrary holds true for hepatitis C virus (HCV), where previous attempts to develop a vaccine were in vain, but novel direct-acting antivirals (DAA) rendered HCV into a curable disease. HCV-associated hepatic and extrahepatic complications of chronic infection, i.e., metabolic syndrome, fibrosis, and HCC, are significantly decreasing in clinical cohorts after sustained virologic response. However, the risk of developing HCC cannot be fully eradicated, especially in patients with advanced liver disease [2,3]. For HBV, a controlled infection by nucleos(t)ide analogues decreases liver disease progression and HCC risk; however, like in HCV-cured patients, a fraction of patients still develop cancer [4,5]. This suggests persistent alterations in the liver, which are induced during chronic infection with HCV or HBV that cumulate until a tip-over, a point of no return for individual patients. Thus for HCV, it is estimated that although viral cure can be achieved in almost all patients treated with DAAs, the peak of HCV-associated liver diseases and HCC may yet to come [6,7]. These facts highlight an important, yet unsolved problem: how can we identify the fraction of cured HCV patients or patients with suppressed HBV viremia that are at still-elevated HCC risk? Moreover, how can we improve the care of these patients that were stratified for closer follow-up with novel clinical strategies, e.g., by HCC chemoprevention? In recent years, it has become clear that only a joint effort of translational scientists and clinicians will be able to provide answers to these eminent questions and develop innovative strategies for reliable biomarkers and novel chemo-preventive strategies aiming at halting liver disease progression and preventing HCC post-HCV cure or in treated HBV patients.

The present Special Issue provides an interdisciplinary view on the current challenges in the post-HCV cure era. It outlines the urgently required next steps to tackle the aftermath of the HCV epidemic to improve the care of HCV-cured patients at risk of developing HCC. Furthermore, this Special Issue reviews the risk of HCC in the treatment of patients with chronic hepatitis B.

First, the HCC risk needs to become measurable in patients using recent advances in omics studies and biomarker development. Naoto Kubota reviews the clinical and molecular determinants available to predict HCC risk including large transcriptional signatures and artificial intelligence [8]. Identified transcriptomic signatures are resulting from underlying virus-induced pro-fibrotic and pro-oncogenic signaling as reviewed by Alessia Virzì [9]. The understanding and mapping of virus-induced signaling and secreted signaling components open new avenues for the identification of minimal-invasive blood biomarkers to identify HCC risk patients. This may help to further increase the reliability of existing biomarkers and avoid liver biopsies. Since signaling and a pro-inflammatory environment can cause metabolic alternations, Chung-Man Moo provides opportunities to exploit hepatic metabolites in normal and cirrhotic livers with and without HCC using proton magnetic resonance spectroscopy with a long echo time [10]. They demonstrated that this method can be used to quantify lactate and triglyceride as well as choline in HCC, which they found elevated in patients with HCC. Along the same line, Silvano Fasolato identified a dysregulated proprotein convertase subtilisin/kexin type 9 (PCSK9), a regulator of lipid homeostasis in HCV-infected livers with and without HCC [11]. It becomes more and more evident that chronic viral infection leaves epigenetic footprints in the host genome, contributing to persistent metabolic changes and cancer risk in patients with suppressed infection or viral cure, as reviewed by Tom Domovitz for HCV [12] and Mirjam Zeisel for HBV [13]. Importantly, some of these changes can be reversed by experimental epidrugs highlighting a potential for new chemo-preventive strategies targeting underlying epigenetic mechanisms. The genetic impact of chronic viral hepatitis results in an evident accumulation of DNA mutations. The genetic damage associated with viral-hepatitis-induced HCC is summarized by Camille Péneau [14]. Beyond the genetic and epigenetic determinants of accumulated and persistent HCC risk are the immunological mechanisms induced by HCV as reviewed by Pil Soo Sung [15] and more specifically for CD8^+^ T-cell responses by Maike Hofmann [16]. What are the clinical challenges and opportunities of patient stratification and the translation of the experimental concepts into practice? Addressing these important questions, Pierre Nahon provides an overview of the current practices and challenges of patient stratification according to HCC risk during HBV control and after HCV cure [17]. Finally, Shen Li’s reviews emphasize secondary prevention strategies of HCC development and its translational challenges [18].

In summary, the articles published within this research topic not only give a highly comprehensive overview of the recent developments and challenges of virus-induced liver cancer risk but also highlight perspectives and opportunities for the discovery and development of biomarkers and chemo-preventive approaches for patients at HCC risk.

## Data Availability

Not applicable.
